# Genetic Effects on the Gut Microbiota Assemblages of Hybrid Fish From Parents With Different Feeding Habits

**DOI:** 10.3389/fmicb.2018.02972

**Published:** 2018-12-04

**Authors:** Wuhui Li, Junmei Liu, Hui Tan, Conghui Yang, Li Ren, Qingfeng Liu, Shi Wang, Fangzhou Hu, Jun Xiao, Rurong Zhao, Min Tao, Chun Zhang, Qinbo Qin, Shaojun Liu

**Affiliations:** State Key Laboratory of Developmental Biology of Freshwater Fish, College of Life Sciences, Hunan Normal University, Changsha, China

**Keywords:** hybridization, reciprocal hybrid, host genetic, gut characteristics, gut microbiota, cellulase content

## Abstract

Gut microbiota play critical roles in host nutrition and metabolism. However, little is known about the genetic effects on the gut microbiota assemblages because a suitable model for investigation is lacking. In the present study, we established the reciprocal hybrid fish lineages derived from the parents with different feeding habits, namely, herbivorous blunt snout bream (*Megalobrama amblycephala*, BSB, 2n = 48) and carnivorous topmouth culter (*Culter alburnus*, TC, 2n = 48). We investigated the genetic effects on gut microbiota assemblages by using 16S rRNA gene sequencing. The results showed that the gut characteristics (structure, relative gut length, relative gut mass, and Zihler’s index) differed between the two types of hybrids and the two parents. In particular, a strong correlation between genotype and gut microbial assemblages indicated that host genetic (subgenome) significantly altered the gut microbial communities. In addition, the microbial structures (composition and abundance) in the two types of hybrids were more similar to those in BSB parent (*P* > 0.05) than to those in TC parent (*P* < 0.05), and the cellulase contents in the gut (produced by gut microbes) also showed the similar results. The results suggested that the host genomic interaction (mainly subgenome domination) had a sizeable effect on shaping the gut microbiota assemblages in reciprocal hybrid fish. This study enriches our understanding of the relationship between host genetic and gut microbiota assemblages, and provides insight into gut microbiota and metabonomics.

## Introduction

Gut microbiotas are among the most densely populated microbial ecosystems. The “gut microbiota” generally refers to the diverse microbial community (bacteria, fungi, archaea, viruses, and protozoa) that colonizes the gastrointestinal tract of its host (Shukla et al., 2017), and this community represents an important enzymatic resource, contributing significantly to food digestion and absorption (Tsuchiya et al., 2008; Ray et al., 2012; Semova et al., 2012; Ni et al., 2014). In recent years, next-generation high-throughput sequencing has shed new light onto the complexity of prokaryotic biodiversity, increasing our understanding of ecological functions between the gut microbiota and its host. Many studies have shown that high microbial diversity levels in the gastrointestinal tract are critical for host nutrition (Hassaan et al., 2017; Li et al., 2017; Mekuchi et al., 2018); disease prevention and immune responses (Milligan-Myhre et al., 2016; Feng et al., 2018); and growth and development (Hill et al., 2016; Egerton et al., 2018).

Fish originated over 600 million years ago and include nearly half of all extant vertebrates, making fish representative of a broad range of physiologies, ecologies and natural histories (Sullam et al., 2012; Wong and Rawls, 2012). Hybridization, a major driving force of speciation and genomic evolution, has been widely applied to aquaculture since the 1980s, as wild-caught fisheries can no longer support the world’s seafood consumption (Mallet, 2007; Gui, 2015; Li et al., 2018). Regarding newly formed hybrid fish, there are more than 32,000 species in the wild (Zhang et al., 2014). Such large fish populations constitute a large spectrum of dietary niches, making them ideal for understanding the evolution and ecology of host-microbiota interactions and host-dietary adaptations (Nayak, 2010; Baldo et al., 2015; Sevellec et al., 2018).

Even though the gut microbiota play critical roles in fish nutrition and metabolism, notably, the composition and abundance, was strongly influenced by many directly and indirectly factors. Many studies showed that gut microbial communities varied according to the host’s ontogenetic development (Bledsoe et al., 2016; Stephens et al., 2016), diet (Bolnick et al., 2014b; Miyake et al., 2015; Hassaan et al., 2017; Zha et al., 2018), environment (Giatsis et al., 2014; Dehler et al., 2017), as well as host genetics and phylogeny (Li J. et al., 2014; Smith et al., 2015; Li et al., 2017; Sevellec et al., 2018). However, previous studies focused primarily on wild-type and capture fish species, such as zebrafish (Roeselers et al., 2011; Semova et al., 2012), carp (Van Kessel et al., 2011; Li et al., 2018), stickleback (Bolnick et al., 2014b; Milligan-Myhre et al., 2016), rainbow trout (Navarrete et al., 2012; Huyben et al., 2018), Catfish (Bledsoe et al., 2018; Zhang and Li, 2018), and whitefish (Sevellec et al., 2018), to investigate the relationship between gut microbes and host physiology, whereas studies on the hybrid fish are rare, especial the reciprocal hybrids fish.

Blunt snout bream (*Megalobrama amblycephala*, BSB, 2n = 48) is an herbivorous freshwater fish with a spindle-shaped body (high and thin), and topmouth culter (*Culter alburnus*, TC, 2n = 48) is a freshwater carnivorous fish with a linearly shaped body (low and long). Both are economically very important freshwater fish with high nutritional value and are widely distributed in the lakes and reservoirs of China. Our previous study obtained an allodiploid hybrid strain (abbreviation BT, 2n = 48) from female BSB × male TC, which successfully generated hybrid F_5_ (Xiao et al., 2014; Zhou et al., 2015). In the present study, we obtained a reciprocal-crossing hybrid (abbreviation TB, 2n = 48) from female TC × male BSB. Interestingly, the two types of hybrids were bisexual fertile, and the divergent genomes merged in somatic cells together presented an ideal model to investigate dietary adaptation and evolution. We comparatively analyzed the intestinal traits, gut microbial structures, and intestinal enzymatic contents of the four groups of fish to evaluate the relationship between genetic differentiation and gut microbiota structure.

## Materials and Methods

### Experimental Fish and Sample Collection

We obtained natural BSB from Liangzi Lake in Hubei Province and natural TC from Dongting Lake in Hunan Province during the month of December. Then, both were fed in a pond with a suitable water temperature and dissolved oxygen content in the Engineering Center of Polyploidy Fish Breeding of the National Education Ministry located at Hunan Normal University, China. During the breeding season (May to June 2015), mature parent BSB and TC (*n* = 5 each) were collected, and self-crossing and reciprocal-crossing were performed as previously described (Xiao et al., 2014). Hybrid BT-F_1_ was from female BSB × male TC, and hybrid TB-F_1_ was from female TC × male BSB. All larvae (approximately 100 each) were divided into two groups (BSB + TC + BT-F_1_ and BSB + TC + TB-F_1_) and fed in the same two-net cage (5 m^2^). One month later, all the fish samples were moved to another larger net cage (20 m^2^), in the same pond.

The fish were fed grass (*Lemna minor*) and artificial fodder (mixed with 45% crude protein, 15% crude fat, 20% crude fiber) routinely (8:00∼10:00 o’clock every day) based on the weather and temperature and occasionally fed with a small amount of live shrimp. Under this condition, the two types of reciprocal hybrids could freely choose food during the feeding process. One and a half years later, we harvested the fish (four groups including BSB, TC, BT-F_1_, and TB-F_1_) for our experiments. All the fish were collected after 8 h of feeding. Before dissection, the appearance, standard body length (BL) and weight (BW), intestinal morphology, and intestinal length (IL) and weight (IW) (*n* = 10 per group) were recorded for each fish with the examination standards referring to “Inspection of germplasm for cultured fishes” of the People’s Republic of China (GB/T 18654.3-2008) (Zou et al., 2008). The intestine of the fish was divided into three parts: the anterior intestine, middle intestine and posterior intestine (Nie and Hong, 1963). Intestinal tissues were excised and photographed, the intestinal contents were carefully packaged to measure enzyme contents, and the remaining posterior intestinal contents were sampled for bacterial 16S rRNA gene sequencing. All the samples were stored in -80°C until used in experiments.

### DNA Extraction and Bacterial 16S rRNA Gene Pyrosequencing

The QIAmp© Fast DNA stool mini kit (QIAGEN) was used to extract DNA according to the manufacturer’s protocol as previously described (Sevellec et al., 2018). Partial DNA fragments of bacterial 16S rRNA genes were amplified by touchdown PCR, as it is the optimal method for avoiding eukaryotic contamination (Korbie and Mattick, 2008). Variable regions (V3∼V4) of the 16S rRNA genes were amplified with a primer pair (515F: 5′-GTGCCAGCMGCCGCGGTAA-3′ and 806R: 5′-GCACTACHVGGGTWTCTAAT-3′). Each sample was amplified in triplicate in a reaction volume of 25 μl containing. The touchdown PCR of bacterial DNA utilized 25 μl of NEBNext Q5 Hot Start Hifi PCR Master Mix, 1 μl (0.2 μM) of each specific primer, 13 μl of sterile nuclease-free water, and 10 μl of DNA (200 ng/μL). The PCR program consisted of an initial denaturation step at 98°C for 30 s, followed by 20 cycles at 98°C for 10 s, 67-62°C (touchdown PCR annealing step) for 30 s, and 72°C for 45 s. After the initial touchdown PCR cycles, an additional 15 cycles were run at 98°C for 10 s (denaturation), 62°C for 30 s (annealing) and 72°C for 45 s (extension), with a final extension of 72°C for 5 min. PCR products (A region ∼ 250 bp in the 16S rRNA gene) were purified using Agencourt Ampure XP beads (Beckman, Brea, CA, United States) according to the manufacturer’s instructions. PCR products were subsequently quantified using the PicoGreen dsDNA Assay Kit (Invitrogen, Carlsbad, CA, United States). Equal amounts of each sample were combined and gel-purified using a QIAquick Gel Extraction Kit (QIAGEN, Valencia, CA, United States) before being re-quantified using PicoGreen. The prepared DNA library was sequenced by MeiJi Biomedical Company (Shanghai, China) using the MiSeq platform (2 × 300 bp, Illumina, San Diego, CA, United States).

### Sequence Data Processing

To achieve taxonomy assignments from 16S rRNA sequence reads, low-quality sequence ends, tags and primers were removed, and sequences were depleted of any non-bacterial ribosome sequences and chimeras using Black Box Chimera Check software (B2C2) (Gontcharova et al., 2010). Then, paired end reads of sufficient length (minimum 20-base overlap between forward and reverse reads) were merged into full-length sequences by FLASH v1.2.5 (Magoc and Salzberg, 2011). Merged sequences (read length > 300 bps, without ambiguous base “N”, and average base quality score > 30) were used for further analysis. The processed reads were clustered into operational taxonomic units (OTUs) with the CD-HIT algorithm using a 97% sequence similarity level by Uclust (usearch v5.2.32) (Edgar, 2010) (Supplementary Table S1).

### Measurement of Enzymatic Content

Intestinal content samples were sent to Lvyuanbode Biotechnology Company (Beijing, China) to measure the intestinal cellulase enzymatic content with an ELISA kit, followed by operation induction. The ELISA kit combined the double antibody sandwich method and color substrate reaction. The changing color and depth were correlated with the enzymatic content. Microplate readers were used to measure the absorbance (OD) value at 450 nm, and the enzymatic content was calculated with a standard curve. All samples were first diluted in a gradient concentration series.

### Statistical Analysis

To determine the significance of differences between microbial communities, Quantitative Insights Into Microbial Ecology (QIIME) 0.8^1^ was used to estimate alpha and beta diversity (Caporaso et al., 2010). Alpha diversity was determined using the Shannon and Simpson indices, and beta diversity indices were calculated using principal component analysis (PCA) by Unscrambler V.9.7 (Camo, Oslo, Norway) as previously described (Caporaso and Gordon, 2011). Redundancy analysis (RDA) and linear regression analysis (LRA) were applied to examine the effects of genetic factors on gut microbiota communities based on the results of PCA (Berenzen et al., 2005). Analysis of similarity statistics (ANOSIM) was estimated using the same Bray-Curtis distance matrix to test the significance of differences between the four groups of fish (Douterelo et al., 2013). The Spearman correlation coefficient was calculated between the four groups of fish to show the effects of genetic factors on average microbiota assemblages by hierarchical clustering. A ternary plot was used to evaluate the proportions of and relationships between the microbiota of the hybrids and the two parents (Takada et al., 2018). PICRUSt was used to predict the potential function of the OUT sequences against a database of 16S bacterial sequences from the National Center for Biotechnology Information (NCBI), including KEGG^2^ and COG^3^ (Langille et al., 2013).

Relative gut length (RGL = IL/BL), Zihler’s index [ZI = IL(cm) × BW(g)^1/3^], relative gut mass (RGM = IW/BW), and relative gut density (RGD = IL/IW, cm/g) were used to evaluate the effects of ontogeny and diet on the gut dimensions. One-way ANOVA and a two-tailed Student’s *t*-test were used to assess differences in intestinal bacterial communities between the four fish groups. Statistical analyses were performed using SPSS 18.0 software (IBM, New York, NY, United States). Raw read sequences of the 16S rRNA gene from fish gut-associated microbiota in this study are publicly available in the NCBI SRA depository within BioProject PRJNA415671, with BioSample accession numbers SAMN07965882-SAMN07965913.

## Results

### Genetics Differentiated Between the Reciprocal Hybrid and Two Parents

**FIGURE 1:**
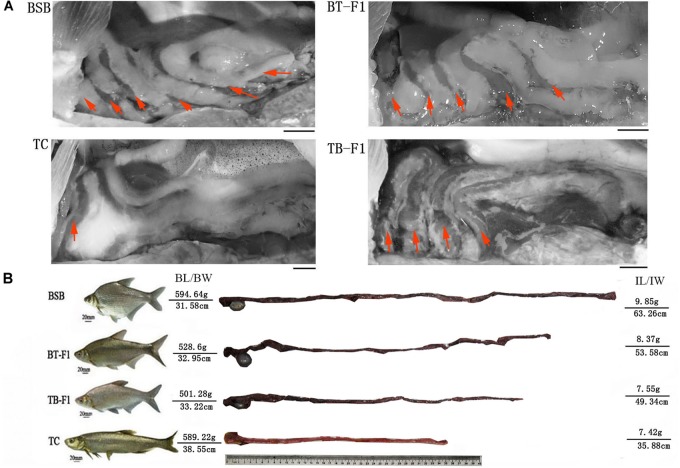
Gut structures of reciprocal hybrid fish and their parents. **(A)** Gut structures of the BSB and TC parents and the BT-F_1_ and TB-F_1_ hybrids; bar = 10 mm. **(B)** Relative gut lengths of the BSB and TC parents and the BT-F_1_ and TB-F_1_ hybrids, both ends showed the fish phenotype, body length and body weight, gut length and gut weight of fish individuals. Red arrows showed the intestinal-loop times.

First, we comparatively analyzed the gut characteristics of the two types of hybrids and parents to evaluate genetic diversity and genomic interactions. We observed that the intestinal structures and morphologies differed markedly between these four groups of fish. The herbivorous BSB possessed an intestinal convolution with a “six-loop” pattern, showing a more complex intestinal structure than that of the carnivorous TC (a simple “one/two-loop” intestine). Interestingly, the two hybrid types, namely, BT-F_1_ and TB-F_1_, exhibited intestinal convolution with a “four/five-loop” pattern and a “four-loop” pattern, respectively (Figure 1A). Our results showed that the herbivorous BSB possessed the longest intestinal structure, and the carnivorous TC exhibited the shortest structure, with the lengths of the two types of hybrids falling in between those of the two parents (Figure 1B).

Several parameters, including RGL, ZI, RGM, and RGD, were used to estimate the effects of ontogeny and diet on the gut dimensions. RGL, ZI, RGD, and RGM were statistically higher in the herbivorous BSB parent than in the carnivorous TC parent (*P* < 0.05, one-way ANOVA) (Table 1). However, no significant difference was detected between the two types of hybrids. Notably, the four parameters of the two types of reciprocal hybrids were closer to those of the BSB parent than to the TC parent. For example, the RGL of BT-F_1_ (1.73 ± 0.15) and TB-F_1_ (1.68 ± 0.06) were statistical higher than parent TC (0.92 ± 0.03) and more closer to parent BSB (2.26 ± 0.33) (Table 1).

**Table 1:** Summary of basic measurements for two hybrids and their parents.

Samples	RGD	RGM	RGL	ZI
BSB	6.68 ± 0.71	0.018 ± 0.002	2.26 ± 0.33	8.51 ± 1.36
TC	5.21 ± 0.56	0.012 ± 0.001	0.92 ± 0.03	4.23 ± 0.19
BT-F_1_	6.19 ± 0.43	0.016 ± 0.003	1.73 ± 0.15	6.99 ± 0.38
TB-F_1_	6.17 ± 0.77	0.016 ± 0.002	1.68 ± 0.06	6.81 ± 0.62


*RGD, relative gut density; RGM, relative gut mass; RGL, relative gut length; ZI, Zihler’s index. (*n* = 10 per group).*

### Alpha and Beta Diversity Analysis

A combined total of 582,266 16S rRNA gene sequences (252.32 Mbp) was generated, including 561,768 sequences (96.48%) representing a total of 661 effective OTUs. These OTUs were assigned to 10 phyla, 21 classes, 47 orders, 79 families, 121 genera, and 152 species (at least 1 OTU per individual, Supplementary Data Sheet S1).

**FIGURE 2:**
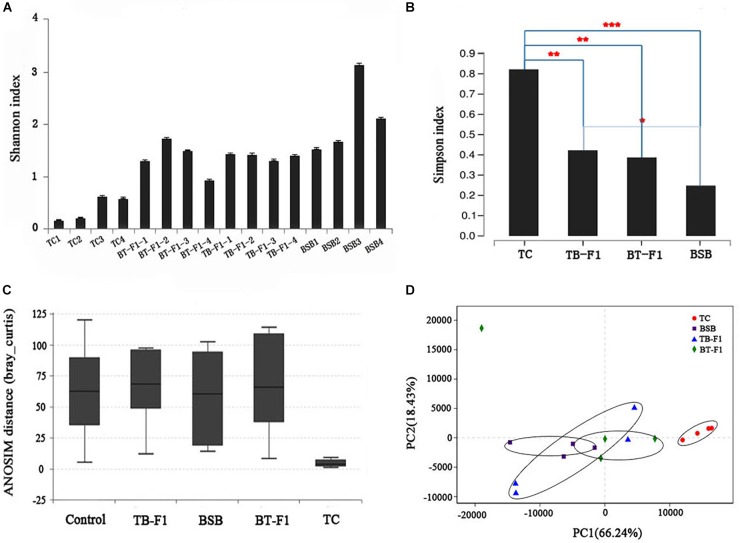
Diversity analysis of gut microbiota between reciprocal hybrids and parents. **(A)** Alpha diversity (Shannon index) was estimate at the genus level. **(B)** Alpha diversity (Simpson index) was estimate in the four groups of fish samples. **(C)** Beta diversity (ANOSIM) estimate at the genus level in the four groups of fish samples. **(D)** Beta diversity (PCA) estimates for all microbial taxa in all individuals at the phylum level; the two kinds of hybrids and BSB are grouped together. Both alpha and beta diversity analysis showed the two types of hybrids was bias toward parent BSB than that parent TC. ^∗^Significant difference (^∗∗^*P* < 0.05, ^∗∗∗^*P* < 0.001, Student’s *t*-test).

Alpha diversity was calculated using the Shannon and Simpson indices at the genus level of the four group fish. The average diversity of TC parent (average *P* = 0.37 ± 0.12, *n* = 4) was lower than that of BSB parent (average *P* = 1.82 ± 0.28, *n* = 4), differing significantly between the two fish (*P* < 0.05, Student’s *t*-test), indicating the herbivorous showed a higher biodiversity than carnivorous. Alpha diversity did not differ between hybrid TB-F_1_ (average *P* = 1.12 ± 0.34, *n* = 4) and BT-F_1_ (average *P* = 1.39 ± 0.16, *n* = 4) (*P* > 0.05, Student’s *t*-test) (Figure 2A). In addition, both types of hybrids showed statistically higher diversity than the carnivorous TC parent (*P* < 0.05, Student’s *t*-test) (Figure 2B). The ANOSIM confirmed that beta diversity was significantly different between the four groups of fish (phylum level: *R* = 0.293 and *P* = 0.006; genus level: *R* = 0.284, *P* = 0.008) (Figure 2C). PCA analysis at the genus level revealed that the gut microbiota of all 16 individual fish could be broadly classified into two main clusters. The two types of hybrids and the herbivorous BSB parent were roughly grouped together, and the four individual TC were grouped together (Figure 2D). Together, the two types of reciprocal hybrids showed higher biodiversity than the TC parent (*P* < 0.05), whereas no significant difference was detected between the two types of hybrids and the BSB parent, indicating the two types of hybrids maybe shared a similar gut environment.

### Correlations Between Genetic Factors and Gut Microbiota Assemblages

**FIGURE 3:**
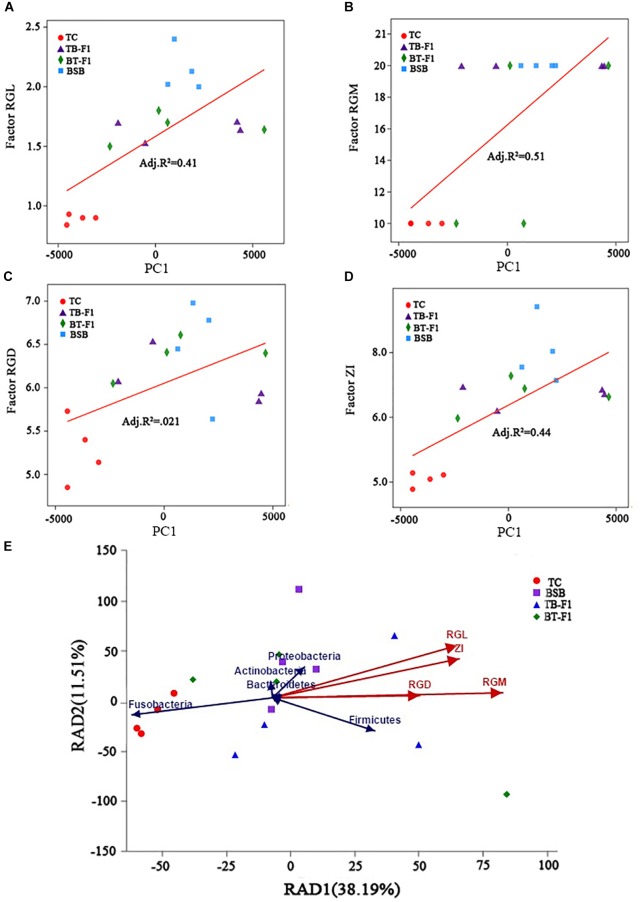
The relationships between intestinal traits and gut microbiota communities. **(A–D)** Linear regression analysis the relationship between the intestinal traits and microbial taxa (principal component, at the OTUs level), RGL **(A)**, RGM **(B)**, RGD **(C)**, and ZI **(D)**; *R*^2^ indicates the squared correlation coefficient, *X*-axis represent the genetics factors, *Y*-axis represent principal component. **(E)** Redundancy analysis (RDA) showed the correlation of genetics factors and dominant microbial taxa. The direction of the color arrow indicates the correlation between intestinal traits and dominant microbial taxa (at the phylum level), red arrows represent genetics factor (intestinal traits), blue arrows represent the dominant microbial taxa in four group fish samples.

**FIGURE 4:**
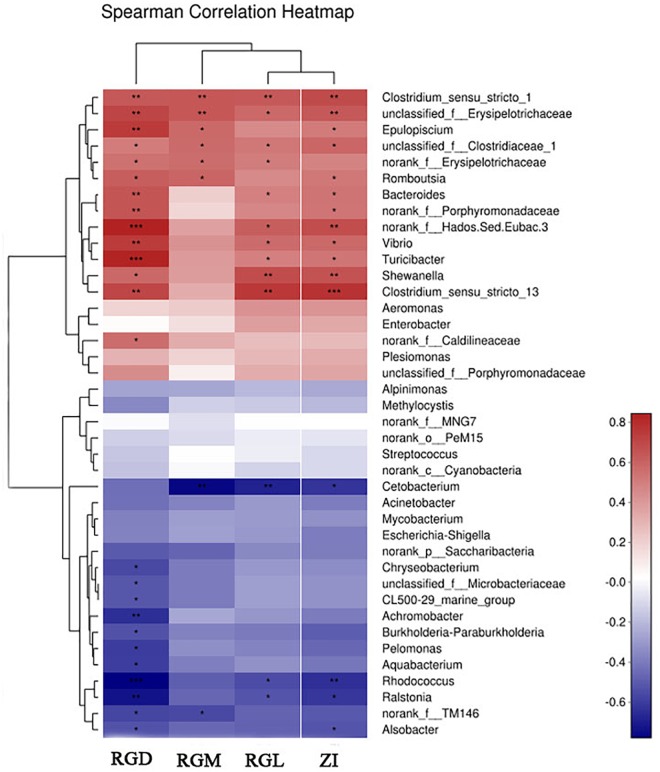
Comparison of microbial classification and gut traits. The Spearman correlation heatmap assesses the correlation between microbial classification (40 dominant genera at the average level) and gut traits. ^∗^Significant difference between the four groups of fish samples, ^∗^*P* < 0.05, ^∗∗^*P* < 0.01, ^∗∗∗^*P* < 0.001 (*n* = 4, Student’s *t*-test).

Because the gut characteristics were different between the two types of hybrids and the two parents, the correlation between genetic factors and gut microbiota assemblages in all fish samples was investigated. First, linear regression analysis (LRA) was based on the genetics factors (RGL, RGD, ZI, RGM) and microbial communities (principal component, at OTUs level). The squared correlation coefficient of LRA was reduced from genetic factors, namely, RGM (*R*^2^= 0.51), ZI (*R*^2^= 0.44), RGL (*R*^2^= 0.41), and RGD (*R*^2^= 0.21) (Figures 3A–D), the higher squared correlation coefficient of gut characteristics (RGM, RGL, ZI) indicating a strong correlation between the genetics factors and gut microbiota taxa. The results of LRA also showed a strong correlation in microbial taxa between the two types of hybrids and parent BSB. Then, RDA analysis showed that the genetic factors greatly shaped the microbiota assemblages in the four groups of fish samples. The dominant microbial taxa at the phylum level, such as Firmicutes, Proteobacteria, Actinobacteria, and Bacteroidetes, were significantly positively correlated with the genetic factors in the two types of hybrids and parental BSB, whereas the microbial taxa Fusobacteria showed a positive correlation with the parental TC samples (Figure 3E). In addition, the results of the Spearman correlation coefficient analysis showed that the positive correlation (*R* > 0.2, *P* < 0.05) was significantly larger than the negative correlation (*R* < -0.5, *P* < 0.05) between the microbiota genus and genetic factors in all fish samples; RGM had the largest effect on microbe abundance, and RGD showed the smallest effect (Figure 4). These results indicated that genetic factors strongly correlated with and greatly shaped the gut microbiota assemblages.

### Composition and Abundance of Shared Bacterial Taxa

**FIGURE 5:**
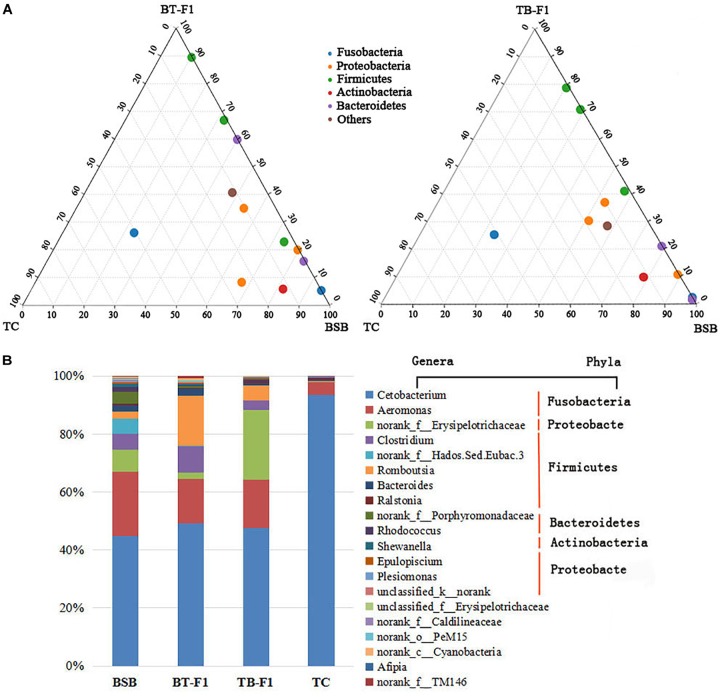
Relative compositions of shared microbial taxa between the two types of hybrids and their parents. **(A)** Ternary plot showed the proportions and relationships of the gut microbial taxa (at the phylum level) between the two types of hybrids and parents, the shared microbial taxa in the two types of hybrids showed strong correlation with parent BSB than parent TC. **(B)** The compositions and abundances of the top 20 dominant genera (assigned to five phyla) in the four groups of fish samples (average abundance, *n* = 4), the two types of hybrids showed a similar tendency with parent BSB than parent TC.

The proportions of shared bacterial taxa between the hybrids and parents were studied because of the high intraspecies variability. The ternary plot showed that the two types of hybrids shared higher proportions of bacterial communities (at the phylum level) with the BSB parent than with the TC parent (Figure 5A). *Cetobacterium, Aeromonas, Romboutsia, Clostridium*, and *Erysipelotrichaceae* were the five most abundant gut bacterial taxa in all experimental fish. Interestingly, the incorporation of the dominant microbiota taxa differed significantly between the herbivorous BSB parent and the carnivorous TC parent. In addition, microbial abundance in the two types of hybrids was more similar to that in the herbivorous BSB parent than to that in the carnivorous TC parent. For example, the incorporation of *Aeromonas* and *Cetobacterium* in hybrids BT-F_1_ (62.41%) and TB-F_1_ (66.45%) was slightly higher than that in BSB (58.22%) but significantly less than that in TC (97.87%) (*P* < 0.05, Student’s *t*-test) (Figure 5B and Table 2). The shared bacterial taxa at the phylum level showed similar results. Fusobacteria and Proteobacteria were the most abundant gut microbiota in the BT-F_1_ and TB-F_1_ hybrids, and the ratios of these bacterial taxa in BT-F_1_ (67.09%) and TB-F_1_ (60.58%) did not differ significantly from that in BSB (61.58%) (*P* > 0.05, Student’s *t*-test) but did differ significantly from that in TC (97.77%) (*P* < 0.05, Student’s *t*-test) (Table 2). The relative compositions and abundances of the microbial genera in all 16 individuals are shown in Supplementary Figure S1.

**Table 2:** Average abundance of dominant gut microbiota in the four fish groups.

	BSB	BT-F_1_	TB-F_1_	TC
				
	Average (%) ± SD	Average (%) ± SD	Average (%) ± SD	Average (%) ± SD
**Phyla**				
Fusobacteria	0.39 ± 0.20	0.49 ± 0.29	0.43 ± 0.26	0.92 ± 0.05
Proteobacteria	0.22 ± 0.06	0.17 ± 0.13	0.18 ± 0.06	0.06 ± 0.06
*Firmicutes*	0.14 ± 0.08	0.30 ± 0.21	0.32 ± 0.11	0.006 ± 0.001
Bacteroidetes	0.06 ± 0.02	0.03 ± 0.02	0.007 ± 0.004	0.002 ± 0.003
Actinobacteria	0.03 ± 0.04	0.03 ± 0.03	0.01 ± 0.01	0.003 ± 0.001

	**BSB**	**BT-F_1_**	**TB-F_1_**	**TC**
				
	**Average (%) ± SD**	**Average (%) ± SD**	**Average (%) ± SD**	**Average (%) ± SD**

**Genera**				
*Cetobacterium*	0.41 ± 0.17	0.47 ± 0.28	0.45 ± 0.28	0.98 ± 0.05
*Aeromonas*	0.17 ± 0.09	0.15 ± 0.13	0.21 ± 0.19	0.05 ± 0.06
*Romboutsia*	0.02 ± 0.01	0.16 ± 0.10	0.10 ± 0.03	0.001 ± 0.001
*Clostridium*	0.04 ± 0.04	0.09 ± 0.10	0.03 ± 0.04	0.003 ± 0.004
*Bacteroides*	0.03 ± 0.04	0.02 ± 0.03	0.007 ± 0.01	0.001 ± 0.001


Functional profiling of microbial communities was predicted based on the 16S rRNA marker gene sequences, as resident/colonized gut microbes may present diverse digestion functions. COG functional clustering showed that most of the 16S rRNA profiles (OTUs) were predominantly associated with host energy production and conversion; amino acid transport and metabolism; and carbohydrate and lipid transport (Supplementary Figure S2). Interestingly, the abundances of enriched COG items increased, in order, from the carnivorous TC parent to the two types of hybrids and to the herbivorous BSB parent. The results indicate gut microbiota not only played critical roles in host metabolism, but also differentiated according to the diet.

### Cellulase Content From Enzyme-Producing Microbes

**FIGURE 6:**
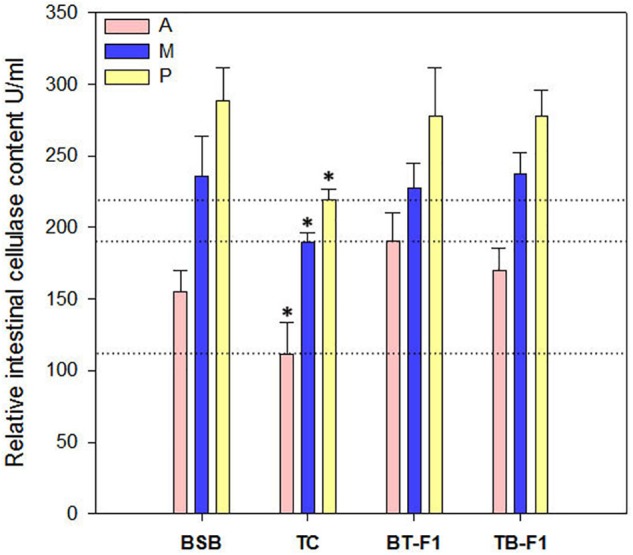
Relative cellulase contents in the two types of hybrids and parents. Cellulase produced by microbial in the intestinal (including Anterior-intestine, Middle-intestine, and Posterior-intestine) was determined in all fish samples by ELISA methods. Cellulase content was increased from Anterior-intestine to Posterior-intestine in all fish samples, and showed a higher level in the hybrids and parent BSB than that in parent TC. A, Anterior-intestine; M, Middle-intestine; P, Posterior-intestine. ^∗^Significant difference in the groups (*P* < 0.05, one-way ANOVA).

Enzymatic activity indicates potential digestive ability. Therefore, the cellulase content was measured in the intestines (anterior, middle, and posterior) of all experimental samples. Cellulase in the two types of hybrids and the herbivorous BSB parent was more active than that in the carnivorous TC (*P* < 0.05, one-way ANOVA) (Figure 6), which is consistent with the proportion of cellulase-producing microbiota (Supplementary Table S2). In particular, the cellulase content gradually increased from the anterior intestine to the middle intestine and to the posterior intestine in all individuals, which indicated that the colonized microbes not only produced cellulase but also increased in number from the anterior to the posterior intestine.

## Discussion

The common assumption is that herbivorous fish possess relatively long narrow intestines that are coiled and fairly uniform in structure throughout, while the intestines of carnivorous species are much shorter, thicker, and straighter, with a greater degree of mucosal folding, than those of herbivorous fish (Kramer and Bryant, 1995; Elliott and Bellwood, 2003; German and Horn, 2006). In particular, several previous studies have utilized intestinal traits (structure, relative mass and length) as predictors of the diets of fish (Wagner et al., 2009; Berumen et al., 2011; Davis et al., 2013). In fact, both food resources and feeding habits play important roles in defining the form and function of the vertebrate gastrointestinal tract (Horn et al., 2006; Karasov and Martinez del Rio, 2007; Davis et al., 2011; Karasov et al., 2011); however, the influence of ontogeny and phylogeny may be larger than that of diet on gut dimensions (German and Horn, 2006; German et al., 2009; Davis et al., 2013). In particular, the study of evolutionary and developmental processes underpinning interspecific differences in gut dimensions has been largely neglected. In the present study, we successfully obtained two types of reciprocal hybrids from hybridization of herbivorous BSB and carnivorous TC. Our results showed that the intestinal traits (structure, RGL, RGM, ZI, and RGD) were significantly different between the carnivorous TC and herbivorous BSB parents (Figure 1 and Table 1), in accordance with previous studies (Kramer and Bryant, 1995; German and Horn, 2006). Interestingly, the two types of hybrids were differentiated after the subgenomic merger, and the intestinal traits of the two types of hybrids were in between those of the parents, with a slight bias toward the traits of the herbivorous BSB parent, indicating that the parent BSB genome may dominate in the two types of hybrids.

The coevolution of mammals and their indigenous microbial communities indicates that herbivorous species have a more diverse gut microbiota than carnivorous species (Ley et al., 2008a). The host diet is among the most important environmental factors influencing gut microbiota diversity (Miyake et al., 2015; Eichmiller et al., 2016; Dehler et al., 2017; Mekuchi et al., 2018; Zha et al., 2018); however, multiple dietary components can interact non-additively to influence gut microbial diversity (Bolnick et al., 2014b). Our results showed that the herbivorous BSB parent and the two types of reciprocal hybrids had statistically higher biodiversity levels than the carnivorous TC parent, even when raised under identical husbandry conditions (Figure 2), indicating that the reciprocal hybrids may have a similar dietary environment to that of BSB. Microbiota diversity may represent functional diversity (i.e., the ability to degrade organic matter), which is not affected by the sampling site but is affected by fish species and diet; genetic diversity is the most significantly influential factor (Mouchet et al., 2012). In fishes, such as eastern bighead carp and paddlefish (Li X.M. et al., 2014), African cichlid fishes (Baldo et al., 2015), Atlantic salmon parr (Dehler et al., 2017), Catfish (Bledsoe et al., 2018) and Asian carp (Eichmiller et al., 2016; Li et al., 2018), studies have demonstrated that Fusobacteria, Firmicutes, and Proteobacteria are the most abundant microbial taxa in the gut. Our data also revealed that the dominant microbiota were Fusobacteria, Firmicutes, Proteobacteria, and Bacteroidetes in the four groups of samples, which is consistent with previous studies. Specifically, microbial structures in the two types of hybrids were more similar to those in the herbivorous BSB parent than in the carnivorous TC parent (Figure 5). Our results suggested that the similar microbiota structure may represent a similar dietary habit, and the different microbial community abundances indicated that selective forces are acting within the host. During evolution, changes in the composition of gut microbiota may lead to shifts in its functions, which may finally influence host nutrition and environmental adaptability (Amato, 2013). In addition, we observed different microbial compositions and abundances among different individuals within the same groups because the microbial structure was affected by many factors (such as gut structure, gut size and even the samples repeat) except for diet or host genetics (Supplementary Figure S1).

Numerous studies have documented that gut microbiota are strongly correlated with host genetics. For example, a variation in gut microbiota is affected by the major histocompatibility class II (MHC) genotypes in three-spine stickleback (Bolnick et al., 2014a), and host sex can interact with dietary factors to affect gut microbial composition (Bolnick et al., 2014c). Fish genotypes co-vary with the gut microbial composition, as more genetically diverse populations exhibit more diverse gut microbiota (Smith et al., 2015). A significant correlation has been detected between the genotypes and microbial compositions of eight fish species (Li J. et al., 2014). In addition, another study showed a minimal effect of host genetics on microbial structure and inferred function in channel catfish and blue catfish (Bledsoe et al., 2018). If a strong correlation between fish genotype and a desirable gut microbial community with beneficial function can be established, this correlation would suggest that the host traits are heritable. In the present study, a strong correlation between gut characteristics and gut microbiota taxa was observed, and the dominant microbial taxa (at the phylum level) were significantly positively correlated with the genetic factors of the two reciprocal hybrids and the two parents (Figure 3). These results indicate that a genomic merge and interaction may have a direct or indirect effect on the fish’s dietary adaptation and evolution and, ultimately, on strongly correlating and greatly shaping the gut microbiota assemblages in cultured reciprocal hybrid fish.

Microflora in the host digestive tract played a significant role in digestion and metabolism (Ganguly, 2012). For example, *Clostridia* in the gut can ferment polysaccharides and proteins to produce alcohols and short-chain fatty acids in mammals (Ley et al., 2008b). *Clostridiaceae* and *Enterobacteriaceae* were identified as active fermenters in the earthworm gut content (Wüst et al., 2011). The cellulolytic bacterial community, including *Aeromonas, Enterobacter, Enterococcus, Citrobacter, Bacillus, Raoultella, Vibrio* and some unclassified bacteria, were dominant in the guts of herbivorous fish (Ray et al., 2012; Li et al., 2016). In our study, we also identified several microbial taxa that contributed to enzyme production in the gut, such as *Aeromonas, Clostridium, Enterobacter*, and *Vibrio*, in all samples. Interestingly, the incorporation of these microbial communities in the herbivorous BSB parent and the two types of hybrids was significantly higher than that in the carnivorous TC parent (*P* < 0.05, Student’s *t*-test) (Table 2 and Supplementary Data Sheet S1). In addition, the intestinal cellulase contents of the two types of hybrids were also similar to those of herbivorous BSB, and showed statistically significantly higher than that of carnivorous TC. The cellulase content corresponded to the incorporation of enzymatic microbial communities, which indicated that the two types of hybrids may have similar diets to that of BSB (Figure 6 and Supplementary Table S2). However, enzymatic activity (content) in fish may be influenced by different exogenous and indigenous factors, such as the age of the fish, type of feed, season and/or temperature of acclimatization, food intake and retention time (German et al., 2004; German, 2009; Jhaveri et al., 2015; Mazumder et al., 2018). Further studies are needed to investigate the exogenous and indigenous factors that influence the enzymatic activity of the hybrids and parents.

In summary, the present study is the first to address the relationship between the host phylogeny, gut microbiota and enzyme content of reciprocal hybrids fish and their parents with different feeding habit raised under identical husbandry conditions. We investigated (1) the gut characteristics of the reciprocal hybrids fish after subgenomic merger and interactions; (2) the gut microbiota structure according to the different feeding habits; and (3) cellulase contents in the gut of all fish samples. Our results showed that the gut characteristics and microbiota assemblages (as well as cellulase produced by microbe) in the reciprocal hybrids were more similar to those of the BSB parent than to those of the TC parent, indicating that the two types of hybrids may have a similar diet to that of the BSB parent. This study sheds new light on the correlations between host genetic characteristics and gut microbiota and provides a perspective on fish dietary adaptation and evolution. Furthermore, understanding the gut microbiota assemblages may provide perspectives on sustainable growth for the aquaculture sector and breeding. However, the detailed functions of the microbial assemblages that change in the gut based on host food digestion and the microstructure of the intestinal tract require further investigation. In addition, more experiments should explore generation F_2_, and the genetics (subgenomic interaction and gene expression) should be considered. Future studies can be extended into analysis at the tissue, cellular and molecular levels to examine the roles of genetics in feeding and metabolism.

## Ethics Statement

This study was carried out in accordance with the recommendations of animal experimentation of the National Research Institute of Fisheries Science, Fisheries Research Agency. The protocol was approved by the Animal Care Committee of Hunan Normal University and Administration of Affairs Concerning Animal Experimentation of China.

## Author Contributions

SL designed the study. WL and CY provided the preliminary data that supported this study. HT and SW performed the daily animal care and laboratory assays. LR and RZ performed the bioinformatics analysis. FH and MT advised on the bioinformatics analysis. JL and QL performed the analyses of the other data. WL and JL wrote the manuscript. JX and QQ provided expert comments. All authors read and approved the final manuscript.

## Conflict of Interest Statement

The authors declare that the research was conducted in the absence of any commercial or financial relationships that could be construed as a potential conflict of interest.

## Footnotes

1 http://qiime.sourceforge.net/

2 http://www.genome.jp/kegg/

3 http://eggnog.embl.de/

## Supplementary Material

FIGURE S1 Relative composition and abundance of microbial genera in all 16 fish samples. Histogram showed the relative abundance of microbiota communities (at genus level) in all 16 individual fish, the detail composition of microbial genera also showed a difference between groups and individuals.

FIGURE S2 Potential function prediction of microbial in the four fish groups. Potential function of microbial (average OTU level) was predicted by search against KEGG database. Histogram results showed the number of OTUs clustered in COG database in the four group fish (*X*-axis represent the COG function item and *Y*-axis represent the average number of OTUs). The most clustered COG item was Amino acid transport and metabolism, indicate the gut microbiota main contribute to host’s metabolism.

TABLE S1 Basic information of the 16S rRNA gene sequences.

TABLE S2 Intestine cellulase content in the four fish groups.

DATA SHEET S1 Basic information of gut microbiota in all fish sample.
