# Supplementary figures and images for: Genetic Effects on the Gut Microbiota Assemblages of Hybrid Fish From Parents With Different Feeding Habits
Source: Front Microbiol. 2018 Dec 4;9:2972. doi: 10.3389/fmicb.2018.02972 (PMC6288232; doi:10.3389/fmicb.2018.02972)

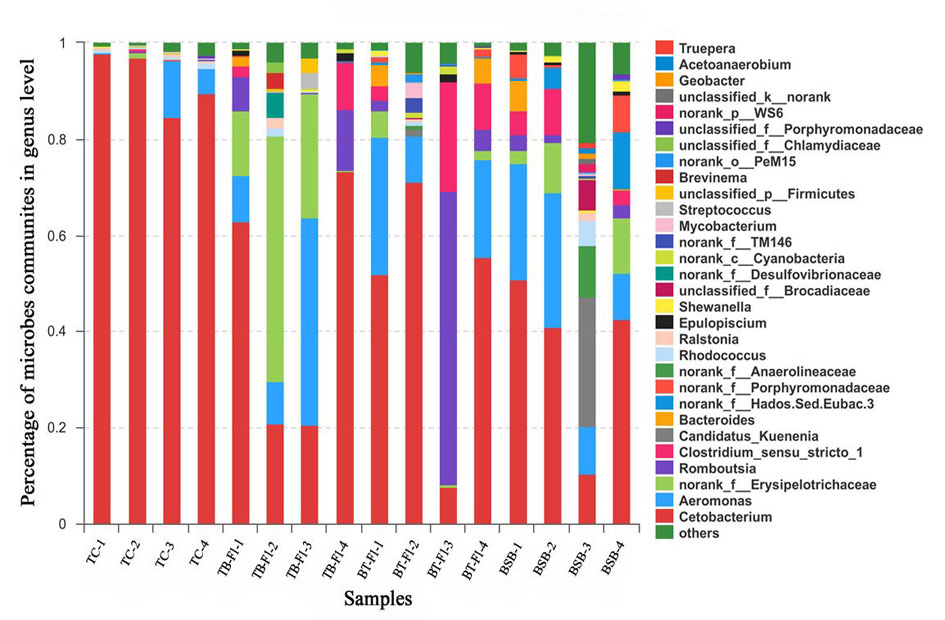

Supplement: FIGURE S1 — Relative composition and abundance of microbial genera in all 16 fish samples. Histogram showed the relative abundance of microbiota communities (at genus level) in all 16 individual fish, the detail composition of microbial genera also showed a difference between groups and individuals. [file Image_1.jpg]

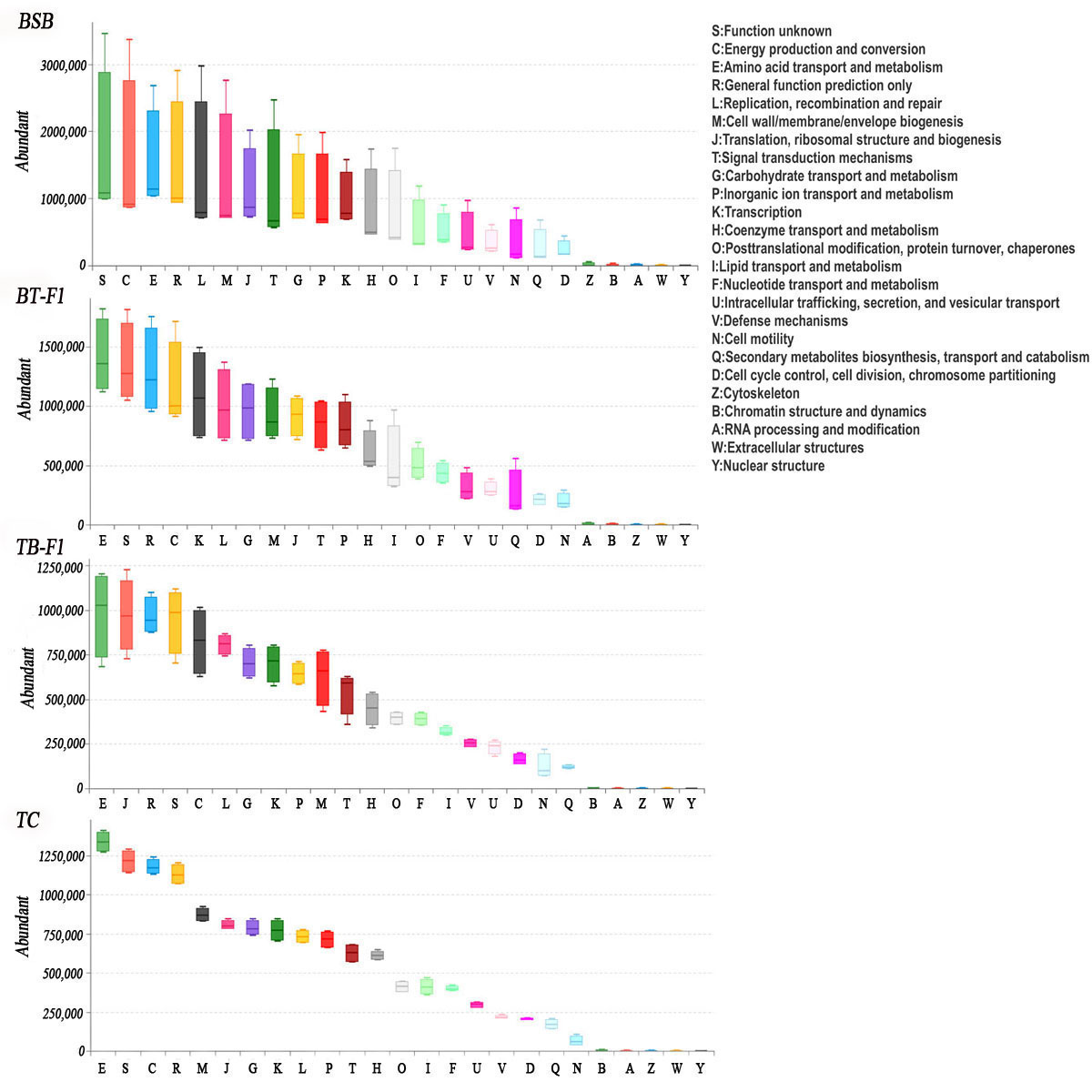

Supplement: FIGURE S2 — Potential function prediction of microbial in the four fish groups. Potential function of microbial (average OTU level) was predicted by search against KEGG database. Histogram results showed the number of OTUs clustered in COG database in the four group fish (X-axis represent the COG function item and Y-axis represent the average number of OTUs). The most clustered COG item was Amino acid transport and metabolism, indicate the gut microbiota main contribute to host’s metabolism. [file Image_2.jpg]
