# Supplementary material for: Genetic Effects on the Gut Microbiota Assemblages of Hybrid Fish From Parents With Different Feeding Habits
Source: Front Microbiol. 2018 Dec 4;9:2972. doi: 10.3389/fmicb.2018.02972 (PMC6288232; doi:10.3389/fmicb.2018.02972)
Supplement: TABLE S1 — Basic information of the 16S rRNA gene sequences. [file Table_1.docx]

**Table S1. Basic information of the 16S rRNA gene sequences**

| Samples | Seq_No. | Base_ No. | Mean_length | OTUs | Effective Seq |
| --- | --- | --- | --- | --- | --- |
| TC-1 | 36,034 | 15,435,261 | 428 | 35,741 | 99.19% |
| TC-2 | 36,985 | 15,940,625 | 431 | 36,535 | 98.78% |
| TC-3 | 36,189 | 15,500,874 | 428 | 35,868 | 99.11% |
| TC-4 | 37,243 | 16,006,164 | 429 | 36,817 | 98.86% |
| TB-F_1_-1 | 38,670 | 16,709,472 | 432 | 36,975 | 95.62% |
| TB-F_1_-2 | 35,682 | 15,170,374 | 425 | 34,851 | 97.67% |
| TB-F_1_-3 | 37,269 | 16,275,540 | 436 | 36,901 | 99.01% |
| TB-F_1_-4 | 37,233 | 16,149,891 | 433 | 34,951 | 93.87% |
| BT-F_1_-1 | 34,081 | 15,172,860 | 445 | 33,685 | 98.84% |
| BT-F_1_-2 | 34,137 | 15,183,182 | 444 | 33,374 | 97.76% |
| BT-F_1_-3 | 37,833 | 16,395,201 | 433 | 37,371 | 98.78% |
| BT-F_1_-4 | 33,356 | 14,251,596 | 427 | 32,165 | 96.43% |
| BSB-1 | 34,811 | 15,342,891 | 440 | 32,876 | 94.44% |
| BSB-2 | 37,668 | 16,421,121 | 435 | 34,918 | 92.70% |
| BSB-3 | 37,498 | 16,332,694 | 435 | 32,782 | 87.42% |
| BSB-4 | 35,403 | 15,459,857 | 436 | 32,299 | 91.23% |
| Total | 582,266 | 252,345,337 | 433 | 561,768 | 96.50% |
