# Supplementary material for: Genetic Effects on the Gut Microbiota Assemblages of Hybrid Fish From Parents With Different Feeding Habits
Source: Front Microbiol. 2018 Dec 4;9:2972. doi: 10.3389/fmicb.2018.02972 (PMC6288232; doi:10.3389/fmicb.2018.02972)
Supplement: TABLE S2 — Intestine cellulase content in the four fish groups. [file Table_2.docx]

| Fish | Anterior-intestine | Middle-intestine | Posterior-intestine |
| --- | --- | --- | --- |
|  | cellulase (U/ml) | cellulase (U/ml) | cellulase (U/ml) |
| BSB-1 | 141.342 | 205.956 | 262.110 |
| BSB-2 | 176.164 | 220.354 | 318.881 |
| BSB-3 | 174.000 | 254.299 | 313.066 |
| BSB-4 | 158.055 | 232.948 | 300.499 |
| TC-1 | 112.531 | 197.704 | 228.543 |
| TC-2 | 132.181 | 186.236 | 212.389 |
| TC-3 | 81.693 | 191.830 | 213.858 |
| TC-4 | 120.341 | 182.480 | 221.619 |
| BT-F_1_-1 | 215.326 | 222.669 | 246.165 |
| BT-F_1_-2 | 184.137 | 224.137 | 274.066 |
| BT-F_1_-3 | 194.767 | 252.039 | 265.255 |
| BT-F_1_-4 | 166.866 | 212.669 | 325.464 |
| TB-F_1_-1 | 153.649 | 235.885 | 263.228 |
| TB-F_1_-2 | 156.307 | 219.173 | 268.011 |
| TB-F_1_-3 | 190.362 | 256.271 | 301.968 |
| TB-F_1_-4 | 178.328 | 238.321 | 267.341 |

**Table S2. Intestine cellulase content in the four fish groups**
